# A high-density genetic map reveals variation in recombination rate across the genome of *Daphnia magna*

**DOI:** 10.1186/s12863-016-0445-7

**Published:** 2016-10-13

**Authors:** Marinela Dukić, Daniel Berner, Marius Roesti, Christoph R. Haag, Dieter Ebert

**Affiliations:** 1 University of Basel, Zoological Institute, Vesalgasse 1, Basel, CH-4051 Switzerland; 2 Centre d’Ecologie Fonctionnelle et Evolutive – CEFE UMR 5175, CNRS – Université de Montpellier – Université Paul-Valéry Montpellier – EPHE, campus CNRS, 1919, route de Mende, 34293 Montpellier Cedex 5, France; 3 Department of Biology, Ecology and Evolution, University of Fribourg, Chemin du Muśee 10, 1700 Fribourg, Switzerland; 4 Biodiversity Research Centre and Zoology Department, University of British Columbia, Vancouver, BC V6T 1Z4 Canada

**Keywords:** Crossover, *Daphnia magna*, Linkage map, RAD-sequencing, Recombination breakpoints, Recombination rate

## Abstract

**Background:**

Recombination rate is an essential parameter for many genetic analyses. Recombination rates are highly variable across species, populations, individuals and different genomic regions. Due to the profound influence that recombination can have on intraspecific diversity and interspecific divergence, characterization of recombination rate variation emerges as a key resource for population genomic studies and emphasises the importance of high-density genetic maps as tools for studying genome biology. Here we present such a high-density genetic map for *Daphnia magna*, and analyse patterns of recombination rate across the genome.

**Results:**

A F2 intercross panel was genotyped by Restriction-site Associated DNA sequencing to construct the third-generation linkage map of *D. magna*. The resulting high-density map included 4037 markers covering 813 scaffolds and contigs that sum up to 77 % of the currently available genome draft sequence (v2.4) and 55 % of the estimated genome size (238 Mb). Total genetic length of the map presented here is 1614.5 cM and the genome-wide recombination rate is estimated to 6.78 cM/Mb. Merging genetic and physical information we consistently found that recombination rate estimates are high towards the peripheral parts of the chromosomes, while chromosome centres, harbouring centromeres in *D. magna*, show very low recombination rate estimates.

**Conclusions:**

Due to its high-density, the third-generation linkage map for *D. magna* can be coupled with the draft genome assembly, providing an essential tool for genome investigation in this model organism. Thus, our linkage map can be used for the on-going improvements of the genome assembly, but more importantly, it has enabled us to characterize variation in recombination rate across the genome of *D. magna* for the first time. These new insights can provide a valuable assistance in future studies of the genome evolution, mapping of quantitative traits and population genetic studies.

**Electronic supplementary material:**

The online version of this article (doi:10.1186/s12863-016-0445-7) contains supplementary material, which is available to authorized users.

## Background

Meiotic recombination is an essential process in sexually reproducing eukaryotes since it is involved in the maintenance of genome stability, in proper segregation of chromosomes into haploid gametes, and in shaping patterns of genetic variation among offspring individuals [1]. Mechanistically, recombination between homologous chromosomes is crucial for accurate repair of DNA double strand breaks that are induced in a highly controlled manner during early meiotic prophase I (reviewed in [1, 2]). Such homology-based repair ensures the maintenance of genome integrity, but also often represents a physical bond between homologous chromosomes, critical for their positioning and proper segregation into the gamete cells [2, 3]. In proceeding meiotic processes, physical connection between homologs will lead to reciprocal (crossover; CO) or unidirectional (gene conversion) exchange of DNA between paternal and maternal chromosomes. It has been shown in several organisms that more abundant and uniformly distributed gene conversions have a limited influence on inherited genetic variation as they affect small genomic regions (350–2000 bp; [4]) On the other hand, CO involves reciprocal allelic exchange across longer chromosomal segments resulting in recombination of genetic variation that can be readily detected following the inheritance of genetic markers in large pedigrees or experimental crosses. Consequently, recombination rate is traditionally approximated as the observed frequency of COs (i.e. neglecting gene conversions) per unit of physical distance (e.g. cM/Mb).

Over the last decade, advancements in sequencing techniques have enabled studies of recombination rate at unprecedented resolution in many different species [5–8]. Importantly, there is accumulating evidence for large amounts of variation in recombination rate across species, populations, individuals, and different genomic regions [5, 7, 9]. This is especially interesting from an evolutionary perspective since the distribution of recombination events across the genome defines the size of genomic fragments that will be incorporated into haplotypes exposed to selection. When recombination is rare, selection wields its influence across long genomic tracts that may contain multiple loci with differing fitness effects. Theory predicts that genetic linkage between multiple sites under selection leads to a reduction of the overall efficiency of selection [10, 11] and high levels of CO recombination are considered favourable for breaking up association between loci subjected to contrasting selective pressures [12]. In addition, genomic regions with low recombination are expected to have lower levels of neutral polymorphism than genomic regions with high recombination rates because of positive (hitch-hiking) or negative (background) selection on sites in their physical neighbourhood [13]. Considering the profound influence that the recombination landscape can have on genome-wide genetic variation and diversity, analysis of the recombination rate emerge as a key resource for population and evolutionary genomics studies, emphasising the importance of high-density genetic maps as essential tools for studying many features of genome biology.

Waterfleas of the genus *Daphnia* have emerged as a well-suited model system for studying genetics of fitness related traits in environmental contexts, due to the extensive knowledge of their ecology, a life-cycle including clonal and sexual reproduction, and the development of genomic resources [14–16]. However, to take full advantage of this model-system, a better understanding of the genome architecture of *Daphnia* is needed, as well as of the mechanisms that are shaping it. In the present study, we use a standard F2 intercross panel and Restriction site Associated DNA (RAD) sequencing for the construction of a high-density genetic map of *D. magna,* one of the best known and most widely used study species of the genus. Including more than 4000 markers across the 238 Mb genome [17], we provide the first characterization of recombination landscape along the chromosomes of *D. magna*. Our data clearly show high levels of recombination towards chromosomal peripheries with chromosomal centres being almost deprived of COs. We discuss these findings in comparison with other organisms and address possible mechanisms underlying the observed patterns of recombination rate variation across the genome of this species.

## Methods

### Design of genetic crosses and DNA isolation

*D. magna* individuals used in this study were obtained by asexually (clonally) propagating lines selected from an F2 intercross panel that had already been used for the construction of microsatellite and SNP-array based genetic maps [17, 18] as well as for QTL mapping [19, 20]. Details about the crossing design can be found in resulting papers. Briefly, the F2 panel was established by first crossing two parental individuals obtained from two inbred clonal lines of *D. magna* (Xinb3 and Iinb1, hereafter referred to as “parental lines”) to produce an F1. One of the parental lines (Xinb3) was a third-generation inbred offspring (three rounds of within-clone mating, each round being genetically equivalent to self-fertilization) of an individual from Southern Finland, the other (Iinb1) was a first-generation inbred offspring of a German individual. A female from the Xinb3 (Finnish mother) and a male from the Iinb1 (German father) parental line were crossed to obtain the F1 hybrid line (called IXF1). By mass-mating genetically identical brothers and sisters of the IXF1 line, F2 offspring were generated, with each initial offspring individual (hatchling from a sexually produced egg) being a founder of a clonal F2 line that was maintained via asexual reproduction as a part of our F2 panel. The Xinb3 line is also the clone on which the *D. magna* reference genome is based (*Daphnia* Genomics Consortium). The draft genome sequence version 2.4 was used in the present study. Finally, two to three females from each parental line, the IXF1 line, and 66 randomly chosen F2 lines were used to establish asexually propagated sub-lines that were used for DNA extractions (pooling nine individuals for each line).

Prior to DNA extractions, all individuals were cleaned by an antibiotic and starvation treatment to minimize algal and bacterial contamination in the sample of genomic DNA. Animals were kept without food during 3 days in a medium containing Ampicillin (Sigma), Streptomycin (Sigma) and Tetracycline (Sigma) at a concentration of 50 mg/L each, and transferred daily to fresh antibiotic medium. To enforce the cleansing of gut contents, a small amount of superfine Sephadex ® G-25 (Sigma-Aldrich) was added frequently to the antibiotic medium, making dextran beads accessible to *Daphnia* for ingestion and gut evacuation. Animals with clear intestine were sampled and used for DNA extractions. In the majority of cases, DNA was isolated immediately after sampling, but in some instances, animals were stored in 70 % ethanol at −20 °C until extraction. DNA extraction was done using the DNeasy Blood and Tissue kit (Qiagen) including the RNaseA (100 mg/ml; Sigma) digestion step.

### RAD library preparation and sequencing

We prepared libraries for RAD-sequencing [21] adopting the protocol of Etter et al. [22] with modifications according to Roesti et al. [8]. Specifically, 1 μg of genomic DNA from each sample was digested with the *PstI* HF restriction enzyme (NEB) in 50 μl reaction volume, for 90 min. at 37 °C and then heat-inactivated following the manufacturer’s manual. A P1 sequencing adapter (5 μl of 100 nM stock solution), containing a unique 5-bp barcode, was ligated to each sample using T4 DNA-ligase (NEB, 0.5 μl of 2,000,000 units/mL stock solution) in 60 μl reaction volume for 45 min at room temperature followed by heat-inactivation for 20 min at 65 °C. The total of 70 samples (Xinb3, Iinb1, IXF1 and 66 F2 lines, with one F2 individual replicated twice) were then combined into two pools (one with 30 and one with 40 samples) and sheared using a Bioruptor (Diagenode). The rationale of combining fewer individuals into the first pool, which included the parental, IXF1 and 26 F2 lines (“parental” library), was to ensure higher sequencing depth and genotyping quality for the founder individuals of the F2 panel, thus facilitating the robust identification of informative SNPs for genetic mapping. The second library contained F2 lines exclusively.

DNA fragments in a range of 250–500 bp were selected using agarose gel electrophoresis (1.25 %, 0.5X TBE), purified and blunt-ended (Quick Blunting Kit, NEB). Klenow fragment exo^−^ (NEB) was used to add dA-overhangs, followed by P2 adapter ligation (1 μl of 10 mM stock solution). Products were purified and PCR amplification was done using Phusion High-Fidelity DNA polymerase (NEB). To minimize the probability of PCR error, master mixes for each library were divided into six separate 12.5 μl reactions for amplification (30 s at 98 °C, 17 cycles of 98 °C 10 s, 65 °C 30 s, 72 °C 30 s, then a final extension for 5 min at 72 °C).

The enriched RAD libraries were sequenced on separate Illumina HiSeq2000 lanes using 100 bp single-end sequencing (Quantitative Genomics Facility service platform, Deep Sequencing Unit Department of Biosystems Science and Engineering, ETH-Zurich in Basel, Switzerland).

### Defining genetic markers for linkage mapping

In total 259,580,561 raw 100 bp reads were generated by sequencing (120,336,323 and 139,244,238 reads in the first and the second library, respectively). Overall read quality was inspected using FastQC (Babraham Bioinformatics, The Babraham Institute) confirming that per-base quality score exceeded 30 (with the exception of the last ten bases of parental library). A custom script (available upon request) coded in R [23] was used to sort raw reads according to unique barcodes into individual samples. Reads containing ambiguous bases and reads that did not feature valid barcode or restriction-site sequence were discarded from further analysis (23 % of the total raw reads). Moreover, the last ten bases were trimmed from the remaining reads due to a decrease in base-calling quality. The cleaned and individually sorted 85 bp reads were aligned to the reference draft genome assembly v2.4 of *D. magna* using Novoalign v2.07 (http://novocraft.com). We allowed on average one high-quality mismatch or indel per 14 bases and accepted only reads that aligned to unique location within the reference genome. Eight F2 samples were discarded because they were sequenced at substantially lower depths compared to the other samples within the same library. In summary, we achieved a mean coverage of 68-fold among the individuals from the parental library (including 22 F2 lines), and 40-fold among the final 37 F2 lines from the second library.

Stacks v1.08 [24] was used for identification of putative marker loci and for genotyping. The samples from the two libraries were analysed separately, taking the differences in sequencing depth into account. In both cases, individual SAM files were imported in Stacks and analysed with the ref_map.pl pipeline. Parental lines were used to construct a “catalog” of loci (3 mismatches allowed between reads mapping to the same locus, option –n in ref_map.pl). The minimum coverage depth (option -m) was set to 25 (parental library) and 15 (lower-coverage library) to call a stack (group of identical reads). Stacks uses error-bounded model for SNP identification however, since the prior information on sequencing error rate was not available, a lower and upper bound for the error rate were not specified (default between 0 and 1). Default chi-square significance level (0.05) required to call a heterozyogote or homozygote was used. Custom MySQL scripts were used for merging the results from both libraries. Deleveraged loci (see [24]), loci with more than three SNPs and loci with more than 2 alleles were excluded from the analysis. In addition, we were only interested in loci that were homozygous for alternative alleles (aa, bb) in the parental lines and heterozygous (ab) in the F1 hybrids. In total, haplotype and genotype data for 7183 putative markers were retrieved from the Stacks analysis.

We inspected the distribution of missing values (per F2 line and per marker) among the obtained genotypes, since they are potential source of errors during linkage map construction. This resulted in the removal of six F2 lines from further analysis because they had more than 30 % of missing genotypes (in comparison, the remaining 52 F2 lines had on average less than 14 % of missing values). Furthermore, we removed markers exhibiting more than 20 % missing data across the F2 lines, as suggested by Catchen et al. [24] and Davey et al. [25]. The resulting dataset comprised 52 F2 lines and 4849 genetic markers in total.

### Linkage analysis

JoinMap 4.0 [26, 27] was used as the main software for genetic map construction. However, several additional steps were taken (Additional file 1: Figure S1) to maximize the number of markers that could be mapped and to avoid the reduction in mapping accuracy that is expected in large datasets (>1000 markers; [28]). First we selected a subset of 253 “anchor” markers (one or two markers per large scaffold that were successfully genotyped in more than 90 % of F2 lines) representing the 211 largest scaffolds of the *D. magna* draft genome assembly (v2.4). Using the regression mapping algorithm with default parameters in JoinMap, these markers were grouped into 10 preliminary linkage groups (LGs) at LOD = 3. Assuming no assembly error at this point of the analysis, all other markers on the same scaffolds were attributed to the same preliminary LG as the respective anchor marker. We then continued to expand the preliminary LGs by performing contingency table analyses of segregation patterns. More precisely, we compared terminal markers of scaffolds that were attributed to one of the preliminary LGs, against the dataset of so-far un-mapped markers. Only markers with very similar segregation patterns (< 3 different genotypes among the 52 F2 lines) were assigned to the same preliminary LG (Pearson’s *χ*2 test, cut-off threshold *P* < 0.0001), whereas markers showing ambiguous association to two or more LGs were discarded at this point of the analysis. Markers with the extreme segregation ratio distortion (SRD) that contradicted surrounding markers within the same scaffold were removed. Following this procedure, 4045 markers were assigned to one of the ten preliminary LGs, 75 markers were removed while 729 markers remained unattributed.

Many markers included in preliminary map showed identical segregation patterns across all F2 individuals (i.e. they did not show any evidence of CO recombination). In total, 756 segregation patterns could be distinguished within our preliminary dataset and the groups of co-segregating markers are hereafter referred to as “bins” (1 to 384 markers per bin). One of the markers exhibiting the lowest number of missing genotypes (i.e., successfully genotyped in the largest number of F2 lines) from each bin was denoted as “frame marker” (unique segregation pattern within the framework map) and was used for creating a framework map, a non-redundant representation of all detected segregation patterns suitable for further analysis with JoinMap. The grouping of frame markers into 10 LGs was confirmed at LOD = 7 (maximum likelihood, ML, option and otherwise default parameter values of the program). We then continued by iteratively adding sets of the remaining, unattributed markers to the preliminary map using same settings in JoinMap. After each round, newly grouped markers were inspected and designated as frame or non-frame markers, depending on whether their genotypes matched one of the previously defined bins. Non-frame markers were continuously omitted from the framework map and kept separately for later construction of a composite map. After several iterations, we managed to include a total of 4761 markers in the composite map while 13 markers did not map to any of the ten LGs and consequently, were omitted from the final dataset. Once all markers were included, the composite map was inspected visually, and the ordering of the markers within the LGs was corrected, based on the available information of physical position within the scaffolds (mostly applying to markers within the same bin, the position of which could not be determined based on segregation patterns). Dubious genotypes were corrected, making the assumption that the vast majority of singletons reflect genotyping errors rather than double CO within short physical distance (i.e. between the focal marker and the adjacent markers on both sides). Thus, if the genotype was not observed in at least 3 adjacent markers within the same scaffold, it was replaced with a missing value [29]. We also checked marker pairs obtained from sister RAD-tags (i.e. markers obtained from RAD loci flanking the same *PstI* restriction site, hence with a distance of <200 bp) and removed one marker of the pair as redundant. If both sister RAD-tags were highly reliable markers (up to three missing values), the consensus segregation pattern was kept (thus reducing the number of missing genotypes in the data set). If the RAD-tag pair showed inconsistent genotypes within the same F2 individual, these instances were replaced with missing values as it is highly improbable that a recombination event happened within such a short distance.

The final composite map comprised 4037 markers, out of which 952 were defined as frame markers (952 bins with 1 to 354 markers). Grouping and ordering of markers within the framework map was confirmed using the ML algorithm at the LOD = 7 (JoinMap, default settings). Afterwards, each LG was analysed individually in JoinMap, with markers in fixed order and genetic distances were calculated using the Kosambi mapping function (Additional file 1). Furthermore, the mapping quality of the framework map was validated through an independent approach using the CheckMatrix program (http://www.atgc.org/XLinkage/).

### Estimating physical distances between markers

The current version (v2.4) of the *D. magna* genome is a still unfinished draft version. Hence, we used the following procedures to estimate the physical distances between markers and the cumulative physical length of each LG: (i) Mapped scaffolds were considered oriented if they had two or more markers separated by at least one recombination event (so the orientation of the scaffold ends could be estimated). Within oriented scaffolds, the distances between markers were known from their alignment position while distances between two terminal markers of adjacent scaffolds were calculated based on the position of markers within their scaffolds and the number of remaining base pairs up to the scaffold’s ends. Note that this assumes no gaps between adjacent scaffolds (see below). (ii) Scaffolds and contigs with only one marker or without detected recombination events were designated as un-oriented. Physical lengths of un-oriented regions were estimated based on the sum of the total lengths of scaffolds and contigs included in those regions. Distances between markers within the non-recombining region were attributed an average value (estimated physical length divided by the number of segments defined by markers). This was done because it was unknown which end segment was adjacent to the next oriented scaffold. (iii) When small contigs mapped inside a longer, oriented scaffolds, their size was not considered, as it was assumed that these contigs mapped to the region of uncertain nucleotides (Ns) inside the scaffold. Such regions are present on all scaffolds due to paired-end sequencing with long, un-sequenced inserts.

### Analysis of recombination rates

R/qtl (countXO function, [30]) was used to count the recombination breakpoints observed in each F2 for each LG. Recombination breakpoints were detected as a change in a genotype along the LGs. More precisely, observed genotype transitions A → H, H → A, B → H or H → B were counted as a single recombination breakpoint, while double breakpoints between successive markers would appear as A → B or B → A genotype transitions (“A” being homozygote for the alleles from the German father clone, “B” is homozygote for the alleles from the Finnish mother clone while “H” annotates heterozygote genotype). The mean number of recombination breakpoints observed in F2 offspring corresponds to the expected mean number of COs during meiosis, averaged across males and females.

Genome-wide recombination rate (GWRR) was calculated by summing cumulative genetic distances of all LGs and dividing it by the most recent estimate of the total length of the *D. magna* genome (238 Mb; [17]). An average recombination rate for each LG (chromosomal recombination rate) was estimated in the same way but we used the physical length that was based only on scaffolds included in our map (see above). We calculated the intra-chromosomal (local) recombination rate between each pair of adjacent markers as the ratio of genetic distance and estimated physical distance between those markers (cM/Mb; Additional file 2). Marey maps were used to plot genetic distance (in cM) against physical distance (in Mb) and to visualise variation in recombination rates along LGs [31]. In addition, local recombination rates were plotted against the physical midpoints of marker intervals, and LOESS (locally weighted scatterplot smoothing) was used for smoothing the estimated values (polynomial degree = 1, α value was adjusted to the density of markers in each linkage group to cover approximately 2 Mb windows). It is important to note here that the chromosomal and the intra-chromosomal recombination rates are probably overestimates because the mapped scaffolds of the reference genome assembly do not cover the full genomic sequence of *D. magna* (only 131 Mb in total). This effect is likely to be particularly strong in repeat-rich regions which are not yet assembled. Therefore, the physical distances used here have to be considered as minimum estimates.

### GC content analysis

To test whether the sequence composition is associated with the recombination landscape in *D. magna*, we investigated how GC content correlates with differences in recombination rate. All analyses of the GC content were done using the available reference genome sequence (v.2.4). At the chromosomal scale, we tested for differences in sequence composition of scaffolds found in recombining vs. non-recombining regions: We compared the average GC content of all scaffolds mapping to regions of low recombination with the ones mapping to regions with high recombination, omitting scaffolds found at the borders of these regions. Furthermore, to assess whether the magnitude of recombination rate correlates with GC content in more discrete intervals, we restricted our analysis to recombining regions only. For this, the two longest scaffolds of each LG were selected and the GC content was extracted for each interval between two markers for which local recombination rate was estimated (interval size between 5 and 100 kb, depending on the spacing between markers).

## Results

### Linkage map

**Fig. 1:**
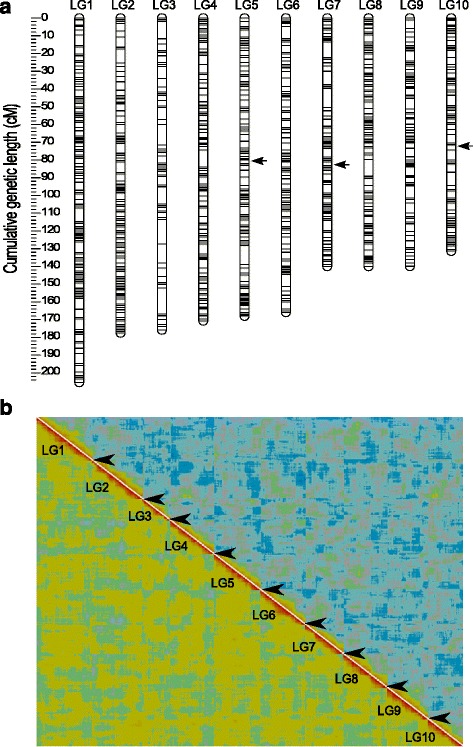
**a** Linkage length and marker distribution of the framework linkage map. The linkage groups (LGs) are ordered from LG1 to LG10 by decreasing genetic length. Only “Frame” markers are shown with *grey lines. Black arrows* indicate regions with segregation ratio distortion (see text). **b** Heatplot as graphical representation of the quality of the linkage map. The image is produced with CheckMatrix (http://www.atgc.org/XLinkage/) to validate the quality of mapping using REC score (*low-left diagonal*) and BIT score (*top-right diagonal*). *Red colour* represents tight linkage and *green to blue colour* indicates no linkage. *Borders* of the LGs are indicated by interruptions (*black arrowheads*) of the red diagonal which confirms the quality of ordering markers within the LG

The genetic map of *D. magna* constructed in this study includes 4037 markers (Additional file 1), assigned to ten LGs that correspond to the ten chromosomes of *D. magna* (*n* = 10). 952 clusters of co-segregating markers (bins) were identified, and only one marker from each cluster was used to assemble a framework map (“Frame” markers; Table 1.). The cumulative genetic lengths (Kosambi corrected) estimated for each LG ranged from 205.4 cM for LG1 to 131.4 cM for LG10, with the total map spanning 1614.5 cM (Table 1). LGs were numbered according to their genetic length estimated in this study (from largest to smallest); not exactly corresponding to the previously published *D. magna* linkage maps [17, 18]. We also note that the terms LG and chromosome are used synonymously throughout the manuscript even though cytogenetic mapping and numbering of chromosomes is not available for *D. magna*. The average genetic distance between frame markers was 1.7 cM with 78 % of the distances being under 2 cM and the largest gap being 14.5 cM (LG3, Fig. 1a), possibly corresponding to a region with a large assembly gap. The independent validation of the framework map is shown as a heatplot (Fig. 1b) with clearly visible LG borders and a red diagonal area, which is generally considered as a sign of high mapping quality (http://www.atgc.org/XLinkage/).

**Table 1:** Linkage map summary. The physical lengths refer to the cumulative length of the scaffolds mapped in each linkage group

Linkage group	Number of markers	Number of “Frame” markers	Genetic length (cM)	Physical length (Mb)	Recombination rate (cM/Mb)
1	441	124	205.38	13.57	15.14
2	706	112	177.51	15.97	11.12
3	312	62	175.77	10.15	17.32
4	449	97	170.68	9.84	17.35
5	426	104	168.02	8.77	19.16
6	407	100	165.91	9.04	18.35
7	377	85	139.98	9.23	15.16
8	362	98	139.96	9.41	14.87
9	319	91	139.89	7.41	18.88
10	238	79	131.38	7.23	18.18
total/average	4037	952	1614.48	100.61	16.55

Three regions showing significant segregation ratio distortion (SRD) were identified. A region spanning 0.77 Mb within LG5 has been described previously, and is due to an allele responsible for the “Unviable Eggs” phenotype [18]. Homozygotes for the alleles from the Finnish mother individual (hereafter B alleles) are highly underrepresented in this region, with complete deficiency located at 80.01 cM (within scaffold00084). Another region, carrying the infertility allele responsible for the “Red Dwarf” phenotype [18] also displayed SRD in our analysis. This region spans approximately 0.69 Mb within LG10 and shows complete deficiency of homozygotes for the alleles originating from the German father individual (A alleles) at 72.29 cM (within scaffold01036). In addition to these previously described regions, we also found a relatively small region with SRD, spanning 0.15 Mb on LG7 (at 81.92 cM). However SRD in this region was weaker than in the two above regions as none of the two homozygotes was completely absent. Nevertheless, the strong deficiency of BB homozygotes in this region (4 % genotype frequency among F2 offspring) suggests the presence of a strongly deleterious, recessive allele in the Finnish mother clone.

### Genome coverage and scaffold mapping

The total size of the *D. magna* genome is estimated at 238 Mb [17]. The draft genome assembly used in this study (v2.4) comprises 40,356 scaffolds and contigs summing up to 131,266,987 bp of genomic sequence (55 % of the estimated genome size). 813 scaffolds and contigs were incorporated in this map (Table 2); this fraction, however, represents 77 % (100,609,459 bp) of the sequence currently assembled and 42 % of the estimated genome size. The high density of markers enabled us to determine the orientation of 97 scaffolds (representing 63,321,641 bp, i.e. 48 % of the reference genome; Table 2). We found only five scaffolds exhibiting inconsistency between the physical position of markers in the current assembly and their segregation pattern. In all instances, these scaffolds comprised two fragments mapping to separate LGs or to different regions of the same LG (Table 3), while the ordering of markers within these fragments remained consistent. These few discrepancies likely indicate errors in the reference genome assembly. Nevertheless, the small portion of scaffolds displaying putative assembly mismatches indicates an overall high quality of the draft genome assembly used here. In addition, scaffold01409 and scaffold01036, spanning parts of the SRD region on LG10 (see above), showed partial overlap, probably due to our inability to precisely map the markers within the region showing SRD.

**Table 2:** Summary of scaffolds and contigs included in the linkage map

Linkage group	No. of mapped scaffolds & contigs	No. of scaffolds	Scaffold bases	No. of contigs	Contig bases	Oriented scaffolds	Oriented scaffold bases
1	84	63	13938534	21	24389	11	10419401
2	112	84	16116820	28	30759	7	9015316
3	81	61	11176544	20	16793	11	2994136
4	73	60	9026485	13	14395	5	6621437
5	102	77	8909170	25	28609	13	5360882
6	81	62	8721446	19	21200	10	6421448
7	72	55	9304851	17	13654	5	6019697
8	72	52	8742755	20	20271	11	5913714
9	77	54	7544014	23	28315	12	5497291
10	59	47	7119613	12	10754	12	5058319
TOTAL	813	615	100600232	198	209139	97	63321641

**Table 3:** Scaffolds of *Daphnia magna* genome assembly v2.4 whose markers map to different linkage groups. An exception is scaffold02227 which is divided into two fragments mapped to different parts of the LG8

Misassembled scaffolds	Total length (bp)	No. markers; position within scaffold	Linkage group
scaffold00093	237880	1 marker; 28344 bp	8
scaffold00093		4 markers; 135580–210844 bp	2
scaffold03387	219786	6 markers; 11029–100849 bp	7
scaffold03387		1 marker; 133916 bp	5
scaffold02486	541490	20 markers; 35514–304827 bp	2
scaffold02486		7 markers; 400767–528088 bp	4
scaffold00233	263417	3 markers; 10570–50454 bp	1
scaffold00233		3 markers; 82320–131292 bp	4
scaffold02227	412371	7 markers; 311258–396970 bp	8
scaffold02227		9 markers; 11932–261034 bp	8

### Recombination rate estimates

**Fig. 2:**
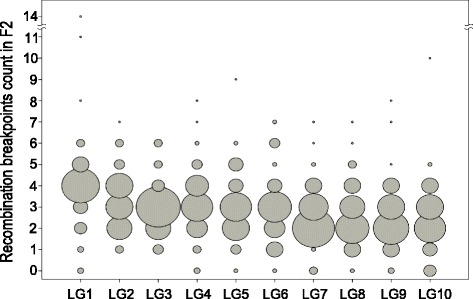
Bubble plot of recombination breakpoints count in each F2 line and LG. *Circle* area corresponds to the number of F2 lines with a specific count

A total of 1564 recombination breakpoints were detected across all F2 individuals and across all LGs. The number of detected recombination breakpoints per individual and LG mainly lies between zero and six, with an average of three and a maximum of 14 (Fig. 2). These counts represent the number of recombination breakpoints observed in F2 offspring, the mean of which also estimates the minimum number of COs that occurred during meiosis in F1, averaged across male and female meiosis. However, the variance in F2 recombination breakpoints and CO numbers during F1 meiosis is not the same, as can be seen from the following consideration: If each chromosome pair undergoes exactly 1 CO per meiosis, 50 % of the resulting gametes will have one recombination breakpoint and the other 50 % will have zero. If these gametes are randomly combined to form F2 individuals, the number of recombination breakpoints in F2 individuals is the sum of those on the two gametes. Hence 25 % of the F2 individuals would have two recombination breakpoints (if each of the two gametes has one), 50 % would have one and 25 % would have zero. Hence, the observation that no recombination breakpoint was observed on some LGs in some individuals (see Fig. 2) does not imply that zero CO occurred in F1 meiosis during gamete formation that gave rise to these individuals.

A genome-wide recombination rate (GWRR) of 6.78 cM/Mb was calculated based on the ratio of the total cumulative genetic map length (1614.48 cM) and the estimated genome size of *D. magna* (238 Mb; [17]). For an estimation of the GWRR based on the genome length that was effectively covered by our markers, we used the total length of the current genome assembly (131 Mb) and accordingly obtained a substantially higher estimate of 12.32 cM/Mb. Due to the gaps within the genome assembly, the later GWRR value has to be regarded as an overestimate.

**Fig. 3:**
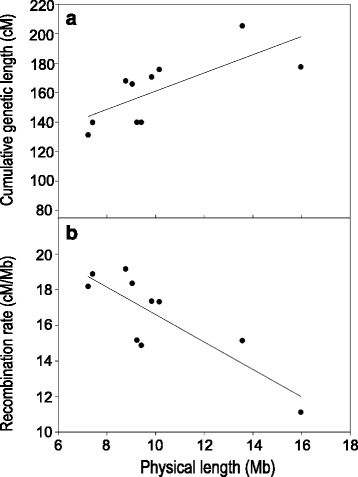
Relationship between estimated physical length of chromosomes and **a** genetic length and **b** chromosomal recombination rate

Nevertheless, assuming that the missing genomic sequence is not randomly dispersed within the genome, but rather uniformly distributed among chromosomes (largely as heterochromatic regions), we can make comparisons between recombination rates estimated for each LG (i.e., chromosome). Genetic length increases linearly with the estimated physical length of each LG (Fig. 3a) with an intercept larger than zero, indicating that even the smallest chromosomes harbour at least one CO. Consequently, smaller chromosomes display more recombination per unit of physical distance resulting in strong negative correlation between recombination rate and the estimated physical length of LGs (Fig. 3b; Pearson’s correlation; *R* = -0.839; *n* = 10; *P* < 0.002).

**Fig. 4:**
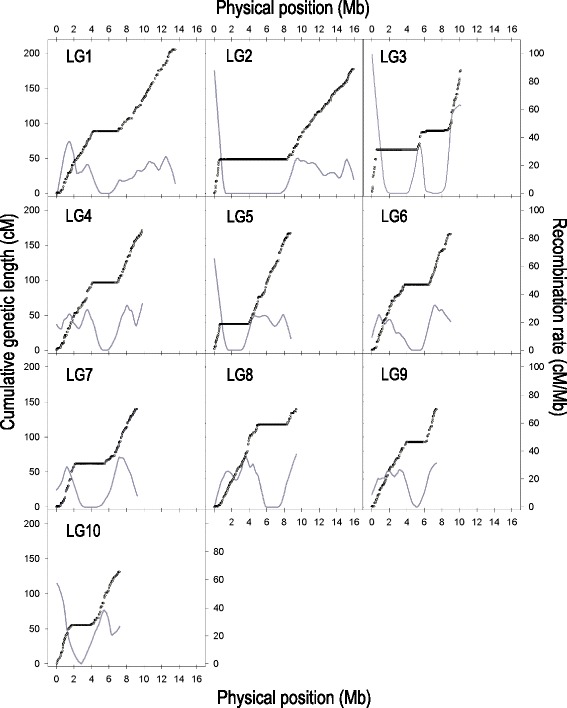
Recombination rate along the 10 chromosomes of *Daphnia magna. Dots* indicate genetic position of markers in centimorgans (referring to the left axis), plotted against their estimated physical position in the genome (in megabases). An average recombination rate (cM/Mb) was calculated for intervals between adjacent markers and plotted against their physical midpoint (Mb). Data points are not shown but the *grey curves* indicate data smoothed by LOESS, with the polynomial degree of one and the sampling portion adjusted for each LG according to the density of markers to obtain a constant smoothing resolution across the panels (moving average of 2 Mb)

Recombination rate varied extensively within LGs (Fig. 4). In each of the 10 LGs, we detected one large region (two in the case of LG3) where recombination was rare or apparently absent. These low-recombination regions are situated mainly in the chromosomal centres and comprise up to 40 % of the mapped genomic sequence. In all cases (except only one of the two regions of LG3), these regions span the map position of the centromere [32]. In each LG the low-recombination regions are flanked by regions of high recombination. Furthermore, we observed a drop in recombination rates towards the very ends of the LGs. However, due to the current state of the genome assembly and the generation of markers sensitive to sequence motifs (RAD), these terminal regions were difficult to study in more detail.

### GC content analysis

We found no difference in the mean GC content between the scaffolds mapping to low-recombination regions and the ones located in regions with high recombination (Paired *t*-test; *n* = 10; *P* = 0.97). Focusing only on scaffolds in highly recombining regions, we found a weak positive correlation between GC content and recombination rate (Pearson’s correlation; *r* = 0.184; *n* = 907 marker intervals; *P* < 10^−8^).

## Discussion

We present a high-density genetic map for *D. magna* that can be coupled with the draft genome assembly, thus providing a valuable resource for genomic investigation and QTL mapping. In contrast to the previously published maps for *D. magna*, all large scaffolds in our map are covered by multiple markers, enabling us to determine their orientation within the chromosome (unless situated in a non-recombining region) and the linkage to other genome segments, which were not previously known. Thus, the linkage map constructed in this way can be used for the on-going *D. magna* genome assembly. Co-segregating markers were used to confirm that the observed patterns of segregation are true biological events rather than methodological artefacts. Hence, although a relatively small number of F2 lines was included in our study, the accuracy of final ordering of markers within and between scaffolds is likely high, much higher compared to previous maps, which were based on few microsatellites [18] or an error-prone SNP-array [17]. In addition to increased reliably, the third-generation linkage map presented here, enables merging of genetic and physical information, and therefore addressing the variation in recombination rate across the genome of *D. magna* for the first time. This is also the most comprehensive study of recombination landscape for any crustacean species reported so far.

### Genome-wide and chromosomal recombination rate

The genome-wide recombination rate (GWRR) of *D. magna* as estimated in the present study is 6.8 cM/Mb, which is slightly higher than the value of 6.2 cM/Mb assessed from the previously published SNP-based map [17]. Similarly, the GWRR of the related species *D. pulex* is estimated at 7.2 cM/Mb [33], suggesting conserved levels of recombination among *Daphnia* species. Much lower GWRRs were reported for a handful of crustacean species for which genetic maps and genome size estimates are available (mean = 1.2 cM/Mb; [34–37]). Also compared to other animal taxa, GWRR of *Daphnia* is high, similar to some Hymenoptera and Lepidopteran species [38]. It has been hypothesized that the elevated GWRRs are favoured in systems with reduced opportunity for sex and recombination including haplodiploidy, cyclic parthenogenesis or species where recombination is restricted to one sex [38]. However, many exceptions from this pattern [36, 38, 39] indicate that peculiar life-cycles *per se* are likely not the only explanation for high recombination rates.

More consistently, it has been shown that recombination rate scales negatively with genome size in many organisms, mainly due to the fact that majority of species have at least one COs per chromosome, even on the smallest chromosomes [40]. Consistent with this, we found that the positive linear relationship between genetic distance and physical distance of chromosomes in *D. magna* has a positive y-intercept, and, hence, smaller chromosomes experience more recombination per physical distance when compared to larger ones.

The mean numbers of observed recombination breakpoints in F2 individuals, as well as the estimated genetic length per chromosome indicate that the different chromosomes of *D. magna* undergo on average between 2.6 and 4.1 CO per meiosis (one expected CO corresponds to 50 cM of genetic map length). It is also interesting to notice that the number of detected recombination breakpoints varies considerably between F2 offspring and individual chromosomes. In 4.8 % of all cases, no recombination breakpoints were detected along an entire LG within a given individual. These instances likely represent the chance union of two gametes that were non-recombinant for this LG. Such gametes occur even in meioses with one or several COs and therefore do not represent evidence for meioses without CO. Furthermore, we may have missed some breakpoints when they were too closely spaced or when they occurred in the terminal chromosome regions, i.e. peripheral to the last marker. However, we believe that this would only explain a small part of the cases without any detected breakpoints. On the other extreme, a few individuals had very high number of recombination breakpoints along a given LG (up to 14). These may suggest a rather high variance in the number of COs per meiosis, or, alternatively, they may partly be explained by genotyping errors. Overall, these instances (in both directions) are, however, rare and hence it is unlikely that they significantly influence the summary statistics on the overall genetic map length presented here.

### Local recombination rates

The genetic map of *D. magna* described in this study revealed major intra-chromosomal variation in recombination rates. The determinants of non-random CO patterning are not yet clear, though several lines of evidence indicate that the hierarchical combination of multiple factors plays a role in shaping the recombination landscape across genomes. These factors include chromosomal size and structural properties, large subchromosomal domains, chromatin structure and the local nucleotide composition [41, 42]. In *D. magna* we found that, CO recombination is more likely to occur in the peripheral parts of the chromosome, while large regions of low or no recombination occur near the central parts of all chromosomes. As for many animal and plant species that were studied earlier [6, 43–46], these regions of extremely reduced recombination coincide with centromeres of *D. magna* [32]. LG3 is an exception because two regions without recombination were detected, though only one of these two regions (the one at 96 cM, also containing a centromere) was also found by Svendsen et al. [32]. The second non-recombining region on this LG might be the result of an inversion or a large indel suppressing recombination specifically in the inter-population cross used for the present study. It is also interesting to note that regions without recombination probably extend to the pericentromeric heterochromatin regions because centromeric regions are usually not included in genetic maps due to the repetitive nature of their sequence (tandem sequence repeats).

Along with the structural confines on recombination landscape, in the majority of animal species that were studied hitherto it has been shown that recombination rates covary with the local nucleotide composition [8, 39, 47–50]. High GC content is considered as a predictor of regions with high recombination rate due to involvement of GC-rich elements in the process of recombination (recognition sites of DNA binding proteins) or, conversely, high recombination rates can lead to high GC content due to GC-biased gene conversions that accompany CO events [51]. In *D. magna* there is no difference in GC content between recombining and non-recombining regions. Within the recombining regions, we found that GC content indeed correlates positively with recombination rate, although the detected correlation is weak. These findings are not surprising considering that the correlation between nucleotide composition and recombination rate occurs at very small physical scales, so testing for this association is strongly dependent on the interval size used and on the precision at which recombination hotspots can be identified.

## Conclusions

Due to the high density of markers included, the genetic map presented here has enabled us to investigate how CO varies in frequency and distribution along the chromosomes of *D. magna.* We have identified large regions of low or no recombination in the chromosomal centres covering approximately 40 % of the mapped genome. These regions also contain the centromeres, but likely extend much beyond the actual centromeric regions. In contrast, CO recombination occurs mainly towards the chromosomal peripheries. These insights into the recombination landscape of *D. magna* can provide a valuable assistance in future studies of the genome architecture, mapping of quantitative traits and population genetic studies. Following improvements in genome annotation, it will be important to understand how gene density correlates with variation in recombination rate. Both the density of loci potentially under selection and the variation in CO rate across the genome can bias genomic analyses and should be considered as important factors in QTL mapping protocols [52] or population genetic studies [13, 51] aiming to understand the effects of selection on genetic variation within and between populations of *D. magna*.

## Additional files

Additional file 1: Figure S1. A flow chart of the genetic map construction process. For a detailed description, see Methods section “Linkage analysis”. (PDF 84 kb)
Additional file 2: The framework and composite map of *Daphnia magna*. Listed information include the names of RAD markers; marker alignment position to *D. magna* genome assembly v2.4; marker assignment as a representative of a segregation pattern (“FRAME” marker), significance level of the segregation ratio distortion (SRD) for each marker based on the *p*-value of Chi-square test for a difference between the observed and the expected Mendelian ratio (*p* < 0.1 *, *p* < 0.05 **, *p* < 0.01 ***, *p* < 0.005 ****, *p* < 0.001 *****, *p* < 0.0005 ******, *p* < 0.0001 *******); the number of the linkage group; the position of the marker within the linkage group (in cM, Kosambi corrected); genotype data used for the construction of genetic map. (XLSX 1401 kb)
Additional file 3: Estimated physical (in bp) and genetic (in cM) positions of all 4037 markers and the average recombination rates (cM/Mb) estimated for intervals between adjacent markers. (XLSX 281 kb)

## Abbreviations

CO: Crossover
LG: Linkage group
QTL: Quantitative trait loci
RAD: Restriction site associated DNA
