# Supplementary figures and images for: A high-density genetic map reveals variation in recombination rate across the genome of Daphnia magna
Source: BMC Genet. 2016 Oct 13;17:137. doi: 10.1186/s12863-016-0445-7 (PMC5064971; doi:10.1186/s12863-016-0445-7)

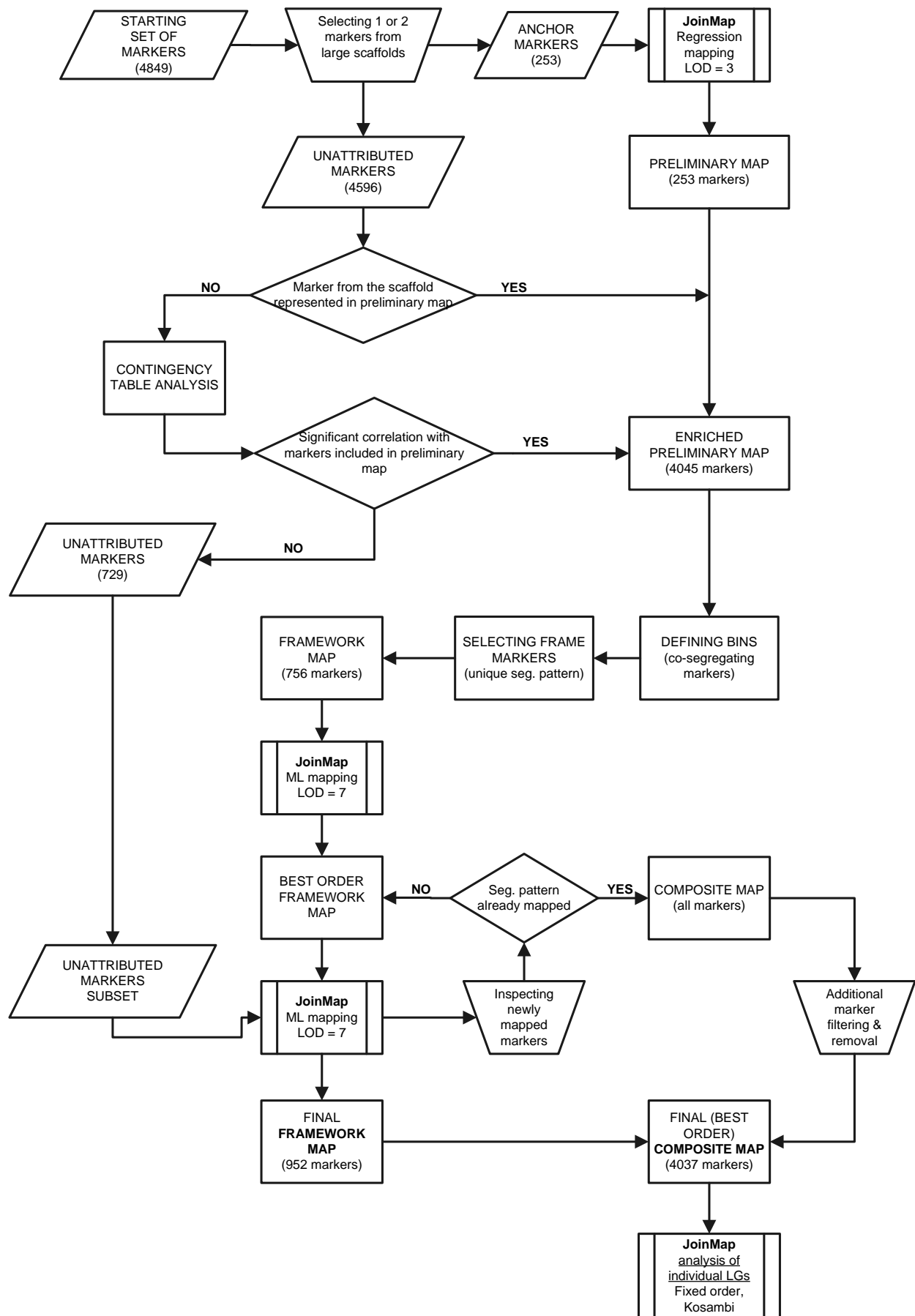

Supplement: Additional file 1: Figure S1. — A flow chart of the genetic map construction process. For a detailed description, see Methods section “Linkage analysis”. (PDF 84 kb) [file 12863_2016_445_MOESM1_ESM.pdf]
